# A Survey of Naturally Occurring Molecules as New Endoplasmic Reticulum Stress Activators with Selective Anticancer Activity

**DOI:** 10.3390/cancers15010293

**Published:** 2022-12-31

**Authors:** Daniela Correia da Silva, Patrícia Valentão, David M. Pereira

**Affiliations:** REQUIMTE/LAQV, Laboratório de Farmacognosia, Departamento de Química, Faculdade de Farmácia, Universidade do Porto, Rua de Jorge Viterbo Ferreira, N° 228, 4050-213 Porto, Portugal

**Keywords:** unfolded protein response, endoplasmic reticulum stress, natural products, chemical library, drug discovery, ATF4, CHOP, EDEM1, caspase-4, calcium homeostasis

## Abstract

**Simple Summary:**

Cancer is the second cause of death worldwide, representing a massive burden on modern society. It is, therefore, urgent to increase the success rates of cancer treatments. With this work, we aim to characterize selectively cytotoxic natural products that act by inducing stress upon the endoplasmic reticulum (ER) of cancer cells. The ER is in charge of the synthesis and folding of most of the cellular proteome. ER stress is a known vulnerability of cancer cells due to their aberrant protein synthesis rates. We identified several natural products that induce selective cytotoxicity on cancer cell lines and act upon the ER. Berberine was the most promising molecule, disrupting Ca^2+^ homeostasis, inducing UPR target gene expression and ER-resident caspase-4 activation on AGS and A549 cells. Our results indicate that berberine and emodin are potential leads for the development of new pharmacological strategies against cancer.

**Abstract:**

The last century has witnessed the establishment of neoplastic disease as the second cause of death in the world. Nonetheless, the road toward desirable success rates of cancer treatments is still long and paved with uncertainty. This work aims to select natural products that act via endoplasmic reticulum (ER) stress, a known vulnerability of malignant cells, and display selective toxicity against cancer cell lines. Among an in-house chemical library, nontoxic molecules towards noncancer cells were assessed for toxicity towards cancer cells, namely the human gastric adenocarcinoma cell line AGS and the lung adenocarcinoma cell line A549. Active molecules towards at least one of these cell lines were studied in a battery of ensuing assays to clarify the involvement of ER stress and unfolded protein response (UPR) in the cytotoxic effect. Several natural products are selectively cytotoxic against malignant cells, and the effect often relies on ER stress induction. Berberine was the most promising molecule, being active against both cell models by disrupting Ca^2+^ homeostasis, inducing UPR target gene expression and ER-resident caspase-4 activation. Our results indicate that berberine and emodin are potential leads for the development of more potent ER stressors to be used as selective anticancer agents.

## 1. Introduction

Recent decades have witnessed an increase in favorable outcomes for cancer patients. There is, however, a long road to achieving high success rates in cancer treatments [1]. In fact, neoplastic diseases remain one of the major causes of death worldwide, second only to cardiovascular disease. Furthermore, the incidence of this type of disease has been increasing [2].

In the eukaryotic cell, protein synthesis and folding, as well as stress response, are inseverable from endoplasmic reticulum (ER) function. This organelle possesses robust signaling networks that have evolved towards the recognition and mitigation of disturbances in proteostasis, which, globally, trigger the unfolded protein response (UPR) [3]. The UPR encompasses three major signaling branches, each activated with one of three leading sensors, namely the (i) protein kinase RNA-like ER kinase (PERK), the (ii) inositol-requiring protein 1 (IRE1), and the (iii) activating transcription factor 6 (ATF6) [3,4,5]. UPR activation decreases the protein load to be processed in the ER lumen by bringing the secretory pathway to a halt. It globally impairs protein synthesis while enhancing the ER-associated degradation of misfolded proteins (ERAD). Whenever it cannot restore proteostasis, UPR activation leads to programmed cell death events [5].

Given their aberrant protein synthesis rates, cancer cells endure chronic ER stress, and their survival strongly relies on UPR activation [6]. Notably, the expression of UPR biomarkers is associated with multiple types of cancers, often implying therapy resistance and poor prognosis [6]. The types of neoplasm in which this has been observed include lung, breast, colon, gastric, pancreas, liver, prostate, kidney, skin, lymphoblastic leukemias, and β-cell lymphoma [7].

One increasingly promising anticancer strategy is the use of molecules capable of triggering ER stress in cancer cells [8]. Relevantly, ER stress inducers often display selective cytotoxic activity against malignant cells, which may be central to the development of safer pharmacological strategies against cancer [9]. When the UPR tolerance threshold is reached in cancer cells, it leads to the occurrence of organized cell death (OCD) [8].

The natural product chemical space, due to its immense structural variability, is a prolific source of ER stress and UPR modulators [10,11,12]. Two remarkable examples of such molecules are thapsigargin (Tg) and tunicamycin, commonly used as research tools to induce ER stress [13,14,15,16,17,18]. Nonetheless, most reports on selective cytotoxic molecules describe cytotoxicity to non-cancer cells, even though they are milder toward cancer cells [19]. Others are reported as selectively cytotoxic due to their specific cytotoxicity towards a few types of cancer cells, but they were not, however, compared to non-cancer cells [20].

Lung and stomach cancers represent a significant fraction of cancer incidence and death worldwide [2]. Here, we report several natural products that are more toxic against a lung cancer cell line than against lung fibroblasts, most of them also being toxic against a gastric cancer cell line. This work aims to report on the effect of cytotoxic compounds against cancer cells among a library of non-toxic natural products that act by triggering ER stress, a known vulnerability of malignant cells [21,22].

## 2. Materials and Methods

### 2.1. Chemical Library and Reagents

The compounds 5,7,8-trihydroxyflavone, 5-deoxykaempferol, apigetrin, coumarin, cyanidin, delphinidin, diosmetin, ellagic acid, eriocitrin, eriodictyol, eriodictyol-7-*O*-glucoside, ferulic acid, fisetin, flavanone, gallic acid, gentisic acid, herniarin, homoeriodictyol, homoorientin, homovanillic acid, isorhamnetin-3-*O*-glucoside, isorhamnetin-3-*O*-rutinoside, isorhoifolin, juglone, kaempferol, kaempferol-3-*O*-rutinoside, kaempferol-7-*O*-neohesperidoside, liquiritigenin, luteolin-3′,7-di-*O*-glucoside, luteolin-4′-*O*-glucoside, luteolin-7-*O*-glucoside, malvidin, maritimein, myricetin, myricitrin, myrtillin, naringenin-7-*O*-glucoside, narirutin, oleuropein, orientin, pelargonidin, pelargonin, phloroglucinol, pyrogallol, quercetin-3-*O*-(-6-acetylglucoside), quercetin-3-*O*-glucuronide, rhoifolin, robinin, saponarin, scopolamine, sennoside B, sulfuretin, tiliroside, verbascoside and vitexin were purchased from Extrasynthese (Genay, France). The molecules (-)-norepinephrine, (+/−)-dihydrokaempferol, 3,4-dihydrobenzoic acid, 3,4-dimethoxycinnamic acid, 3-hydroxybenzoic acid, 4-hydroxybenzoic acid, berberine, betanin, boldine, catechol, cholesta-3,5-diene, cynarin, daidzein, emodin, galanthamine, genistein, guaiaverin, hesperetin, myristic acid, naringenin, naringin, *p*-coumaric acid, phloridzin, pinocembrin, quercetin-3-*O*-β-D-glucoside, quercitrin, rosmarinic acid, rutin, and silibinin were acquired from Sigma-Aldrich (St. Louis, MO, USA). Caffeine was acquired from Fluka (Buchs, Switzerland), vanillin was supplied by Vaz Pereira (Santarém, Portugal), vicenin-2 was purchased from Honeywell (Charlotte, NC, USA). Chlorogenic acid was acquired from PhytoLab (Vestenbergsgreuth, Germany). Cinnamic acid was obtained from Biopurify (Chengdug, China). Swertiamarin was obtained from ChemFaces (Wuhan, China).

Dulbecco’s Modified Eagle Medium (DMEM), Dulbecco’s Modified Eagle Medium/Nutrient Mixture F-12 (DMEM/F-12), fetal bovine serum, penicillin/streptomycin solution (penicillin 5000 units/mL and streptomycin 5000 µg/mL), trypsin-EDTA (0.25%), Qubit™ dsDNA HS assay kit, Qubit^TM^ RNA IQ assay kit, Qubit^TM^ RNA HS assay kit, SuperScript^TM^ IV VILO^TM^ MasterMix, and the Qubit^TM^ Protein Assay Kit were acquired from Invitrogen (Grand Island, NE, USA). Dimethyl sulfoxide (DMSO) was acquired from Fisher Chemical (Loughborough, UK). Isopropanol was obtained from Merck (Darmstadt, Germany). 3-(4,5-Dimethylthiazol-2-yl)-2,5-diphenyltetrazolium Bromide (MTT), calcium ionophore A23187, thapsigargin, RNAzol^®^, chloroform, isopropanol, diethyl pyrocarbonate (DEPC), KAPA SYBR^®^ FAST qPCR Kit Master Mix (2×) Universal, Trizma^®^ base, sodium chloride, potassium phosphate monobasic, potassium chloride, sodium phosphate dibasic, sodium bicarbonate, D-glucose, phorbol 12-myristate 13-acetate (PMA), 4µ8C and Triton X-100 were obtained through Sigma-Aldrich (St. Louis, MO, USA). The fluorescent probe Fura-2/AM and caspase-4 assay kit (fluorometric) were purchased from Abcam (Cambridge, UK). Promega Caspase-Glo™ 3/7 Assay Kit was obtained from Promega Corporation (Madison, WI, USA). Salubrinal and JC-1 iodide were acquired from Santa Cruz Biotechnology (Dallas, TX, USA). Irestatin 9398 was acquired through Axon Medchem (Groningen, The Netherlands).

### 2.2. Cell Culture Conditions

The AGS human gastric adenocarcinoma cell line (American Type Culture Collection, LGC Standards S.L.U., Spain) was cultured in Dulbecco’s Modified Eagle Medium (DMEM) + GlutaMAX^TM^ containing 10% FBS and 1% penicillin/streptomycin. A549 human lung adenocarcinoma cell line (American Type Culture Collection, LGC Standards S.L.U., Spain) was cultured in Dulbecco’s Modified Eagle Medium/F12 (DMEM) + GlutaMAX^TM^ containing 10% FBS and 1% penicillin/streptomycin. Both were maintained at 37 °C with 5% CO_2_. In order to obtain spheroids, the AGS and A549 cells were seeded in hydrophobic round-bottomed 96-well plates in appropriate cell culture medium containing collagen (0.3 µg/mL) at a density of 5 × 10^3^ cells/well. Under these conditions, spheroids were fully formed after 72 h at 37 °C.

### 2.3. Monolayer and 3D Cell Viability Assays

For the determination of cellular viability in the monolayer cell culture, the AGS cells were seeded at a density of 1.5 × 10^4^ cells/well, while A549 were seeded at 1 × 10^4^ cells/well. Afterward, the cells were maintained at 37 °C for 24 h. After this growth period, the wells were incubated with the compounds under study (100 µL/well) for another 24 h. The medium was aspirated and replaced by a fresh medium containing MTT at 0.5 mg/mL. The cells were incubated for 90 (AGS) or 120 min (A549). Finally, the MTT solution was discarded, the resulting formazan crystals dissolved in a 3:1 DMSO:isopropanol solution (200 µL/well) and the absorbance at 560 nm was read using a Thermo Scientific^TM^ Multiskan^TM^ GO microplate reader. All of the experiments were performed in triplicate. The results are presented as the percentage of the control value and correspond to the mean ± SEM of at least three independent experiments.

To determine the viability of cells under 3D culture conditions, the compounds of interest were incubated on the spheroids for a period of 7 days. After this, the Presto Blue^TM^ reagent was added according to the instructions from the manufacturer and incubated for 4 h at 37 °C for both cell lines. Finally, the fluorescence at 560/590 nm was read on a Cytation^TM^ 3 (BioTek, Winooski, VT, USA) multifunctional microplate reader. 

### 2.4. Evaluation of the Mitochondrial Membrane Potential (ΔΨ_m_)

The cells were seeded at the previously referred densities on 96-well plates. Then, 24 h later, they were incubated with the compounds of interest for another 24 h at 37 °C. Afterward, the medium was replaced by HBSS containing the ratiometric probe JC-1 at 7 μM. After 45 min at 37 °C, the wells were carefully washed three times with HBSS. Fluorescence was read at 485/530 nm and at 530/590 nm in a Cytation™ 3 (BioTek) multifunctional microplate reader. The effect upon mitochondrial membrane potential was inferred from the ratio F_530/590_/F_485/530_. PMA at 100 nM was resorted to as a positive control for the disruption of the mitochondrial membrane, while the molecules under study were tested at 50 µM. All of the determinations were performed in triplicate. The results are presented as the fold decrease vs. control, representing the mean ± SEM of at least three independent experiments.

### 2.5. RNA Sample Preparation, Conversion to cDNA and RT-qPCR Analysis

AGS were seeded at 1.2 × 10^5^ cells/well and A549 at 8 × 10^4^ cells/well in 12-well plates. The plates were then kept at 37 °C for 24 h prior to the incubation with the compounds of interest (50 µM) for 16 h. Tg at 3 µM was used as a positive control for upregulated UPR gene expression. The cells were lysed by replacing the culture medium with 500 µL of PureZOL RNA isolation reagent in each well. The lysate was pipetted up and down several times and the PureZOL reagent was transferred to the duplicate well. The process was repeated, and the lysates were maintained at room temperature for 5 min.

The RNA was extracted by phase separation by adding 100 µL of chloroform to each sample and thoroughly mixing, followed by 5 min at room temperature and 15 min centrifugation at 12,000× *g*, at 4 °C. The resulting aqueous phase was transferred to a new tube and mixed with 250 µL of isopropyl alcohol. Again, the mixture was left to stand for 5 min at room temperature and then centrifuged at 12,000× *g* for 10 min at 4 °C. The supernatant was discarded, and the RNA pellet was washed with 500 µL of 75% ethanol and centrifuged for 5 min at 7500× *g* (4 °C). Finally, the supernatant was again discarded, and the pellet was air-dried for a few minutes and resuspended in 25 µL of DEPC-treated water. 

RNA quantification was performed using a Qubit^TM^ RNA IQ assay kit, and its integrity was assessed using the Qubit^TM^ RNA IQ assay kit according to the instructions provided by the manufacturer. The conversion to cDNA was performed with the SuperScript^TM^ IV VILO^TM^ MasterMix, using 1 µg of the RNA sample. The primers (Table 1) were designed on Primer BLAST (NCBI, Bethesda, MD, USA) and synthesized by Thermo Fisher (Waltham, MA, USA). The RT-qPCR reaction was performed with the KAPA SYBR^®^ FAST qPCR Kit Master Mix (2×) Universal in the following thermal cycling conditions: 3 min at 95 °C, 40 cycles of 95 °C for 3 s, gene-specific temperature for 20 s (specified on Table 1), and 20 s at 72 °C. The reactions took place in a qTOWER3 G (Analytik Jena AG, Germany), and the results were viewed on the software supplied with the equipment (qPCRsoft 4.0). *GAPDH* was selected as a reference gene for expression normalization. The results correspond to at least four independent experiments, and every reaction was performed in duplicate.

### 2.6. Cytosolic Ca^2+^ Level Determination

The AGS and A549 cells were plated at the aforementioned densities on black-bottomed-96-well plates and incubated at 37 °C for 24 h. Then, the medium was replaced by the fluorescent probe Fura-2/AM at 5 µM in HBSS and incubated for 1 h. The molecules under study (50 µM) were then prepared in HBSS and added to the plate. Two hours later, the wells were washed three times with HBSS, and the fluorescence was read at 340/505 and 380/505 on a Cytation^TM^ 3 (BioTek) multifunctional microplate reader. The data analysis was conducted considering the ratio F_340/505_/F_380/505_. Each experiment was performed in triplicate. The results are presented as fold increase vs. control and represent the mean ± SEM of at least three independent experiments.

### 2.7. Caspase-3 and -4 Activities

To determine the activity of caspase-4, the cells were plated in 6-well plates at 2.4 × 10^5^ cells/well for AGS cells and 1.6 × 10^5^ cells for A549 and incubated at 37 °C overnight. The compounds of interest were then added at 50 µM, and the cells were incubated for 6 h. After this period, the cells lysates were prepared in the lysis buffer provided by the manufacturer of the caspase-4 assay kit, using a volume of 50 µL for each sample. The protein was quantified resorting to the Qubit^TM^ Protein Assay. The assay was conducted in black-bottomed 96-well plates, using 100 µg of protein for each determination. The protein samples were incubated with the reaction buffer and the caspase-4 substrate LEVD-AFC at 50 µM for 2 h at 37 °C and, finally, the fluorescent signal was read at 400/505 nm in a Cytation^TM^ 3 (BioTek) multifunctional microplate reader. The experiments were individually performed in duplicate, and the results represent the mean ± SEM of at least three independent experiments.

The activation of caspase-3 was evaluated using the Caspase-Glo^®^ 3/7 kit assay. The cells were seeded, as described above, on white bottomed-96-well plates. After 24 h elapsed, the cells were incubated with the molecules under study for a new period of 24 h. Staurosporine at 100 nM was employed as a positive control for caspase-3 activation. Afterward, 60 of the 100 µL of medium contained in each well were discarded and replaced by 40 µL of the Caspase-Glo^®^ 3/7 buffer, containing Caspase-Glo^®^ 3/7 substrate. The plate was incubated for 30 min at 22 °C, and the luminescent signal was measured in a Cytation™ 3 (BioTek) multifunctional microplate reader. All of the measurements were performed in duplicate. The results are presented as fold increase vs. control and represent the mean ± SEM of at least three independent experiments.

The obtained results were normalized for the DNA amounts present after the 24 h incubation with the molecules. After 24 h, the cells were incubated with ultra-pure water at 37 °C for 30 min and subsequently frozen at −80 °C. The DNA was quantified with the Qubit^TM^ dsDNA HS Assay Kit. The caspase activity results were then normalized with the determined DNA amounts.

### 2.8. Study of the Involvement of the PERK and IRE-1 Branches of the UPR on Cell Viability

The cells were seeded as described for the MTT reduction assay and incubated with the toxic compounds (50 µM) in the presence or absence of salubrinal (1 μM), an inhibitor of eIF2α dephosphorylation and, thus, of PERK signaling, irestatin 9398 (5 μM), an inhibitor of the IRE-1 endonuclease activity, or 4µ8C (2 μM), an inhibitor of both endonuclease and kinase activities of IRE-1. Prior to the incubation of the molecules under study in the presence of the mentioned UPR inhibitors, a pre-incubation of 1 h with these inhibitors was conducted. The medium was then replaced by a solution containing the molecule and the inhibitor at the aforementioned concentrations. Cell viability was determined 24 h later via MTT reduction assay.

### 2.9. Statistical Analysis

Statistical analysis was carried out on the GraphPad Prism 8 software. In order to compare the single treatments with the control groups, we employed the unpaired Student’s *t*-test, and values of *p* < 0.05 were considered statistically significant. The Grubbs test was used to detect outliers.

## 3. Results

### 3.1. Cytotoxicity Evaluation

**Figure 1:**
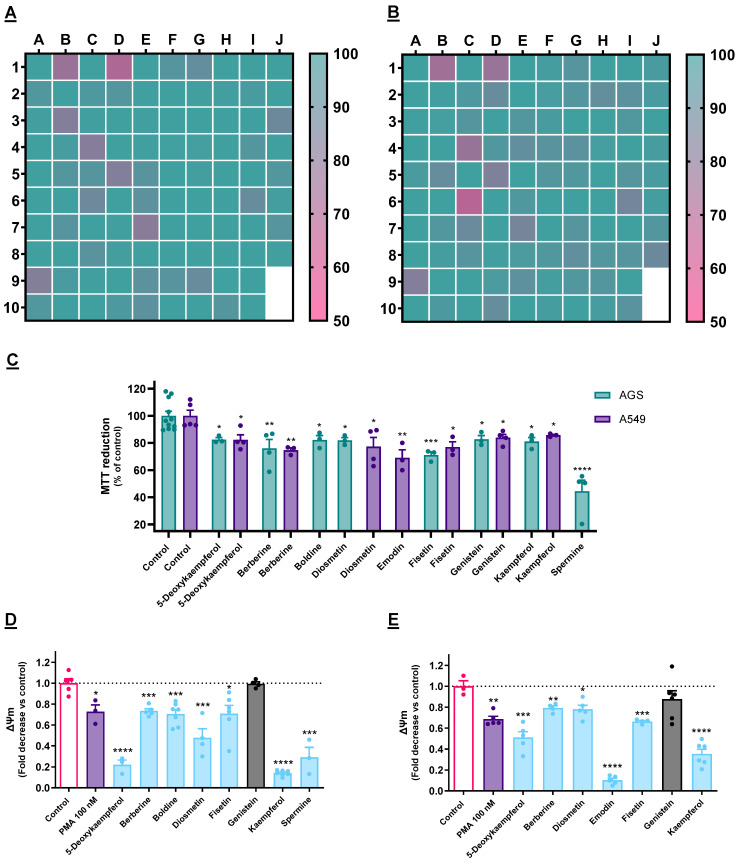
(**A**,**B**): Impact of each compound on the viability AGS (**A**) or A549 (**B**) cells after 24 h, as determined by the MTT reduction assay. Every molecule was tested at 50 µM. Results are presented as the percentage of the control value and correspond to the mean of at least three independent experiments. (**C**): Impact of each of the selectively cytotoxic molecules on the viability of AGS and A549 cells, representing the mean of at least three independent experiments ± SEM. (**D**,**E**): Impact of the selected cytotoxic molecules on the mitochondrial membrane potential (ΔΨ_m_) of AGS (**D**) and A549 (**E**) cells. Results consider F_530/590_/F_485/530_ and express the mean ± SEM of at least three independent assays, all the latter conducted in triplicate. * *p* < 0.05, ** *p* < 0.01, *** *p* < 0.001, **** *p* < 0.0001.

The physico-chemical properties of the compound library used herein, as well as information on the toxicity of the molecules in non-cancer cells, were reported in our previous work [23]. Considering the results obtained with the 97 molecules (Table S1, Figure S1A,B), Figure 1A,B shows the molecules that caused the loss of viability of the two cancer cells lines used, AGS (gastric carcinoma) and A549 (lung adenocarcinoma), at a single concentration (50 µM). Most of the hits revealed toxicity to both cell lines, with three exceptions: boldine and spermine were only toxic to AGS cells, while emodin displayed a cytotoxic effect only towards A549 cells. On the other hand, 5-deoxykaempferol, diosmetin, fisetin, berberine, genistein, and kaempferol were toxic towards both of the studied cell lines (Figure 1C). All of the results are presented in the bar charts in Figures S1 and S2.

As shown in Figure 1D,E, all of the molecules reduced ΔΨ_m_ on both cell lines, with the exception of genistein, which was, for this reason, dropped from the pipeline (Figure 1D,E). It is reported in the literature that PMA causes the disruption of the ΔΨ_m_ at very low concentrations, alongside increased ROS production, by being an agonist of protein kinase C (PKC), as well as inducing its mitochondrial translocation, and hence it was used here as a positive control [24,25]. We have observed that many of our active molecules at 50 µM could exert a stronger effect at this level than PMA, even though the latter was used at 100 nM. This was observed with both kaempferol and 5-deoxykaempferol on both cell lines. Furthermore, emodin displayed a potent effect on the ΔΨ_m_ dissipation in A549 cells.

### 3.2. Effect of Cytotoxic Molecules on UPR Activation

The activation of the UPR involves the increased expression of multiple target genes, representing definitive evidence of stress upon the ER. Representative genes from multiple UPR branches and chaperones (*ATF44*, *HSPA55*, *DDIT3*, *HSP90β1* and *EDEM1*) were selected, and their expression following the treatment with the cytotoxic molecules was studied.

**Figure 2:**
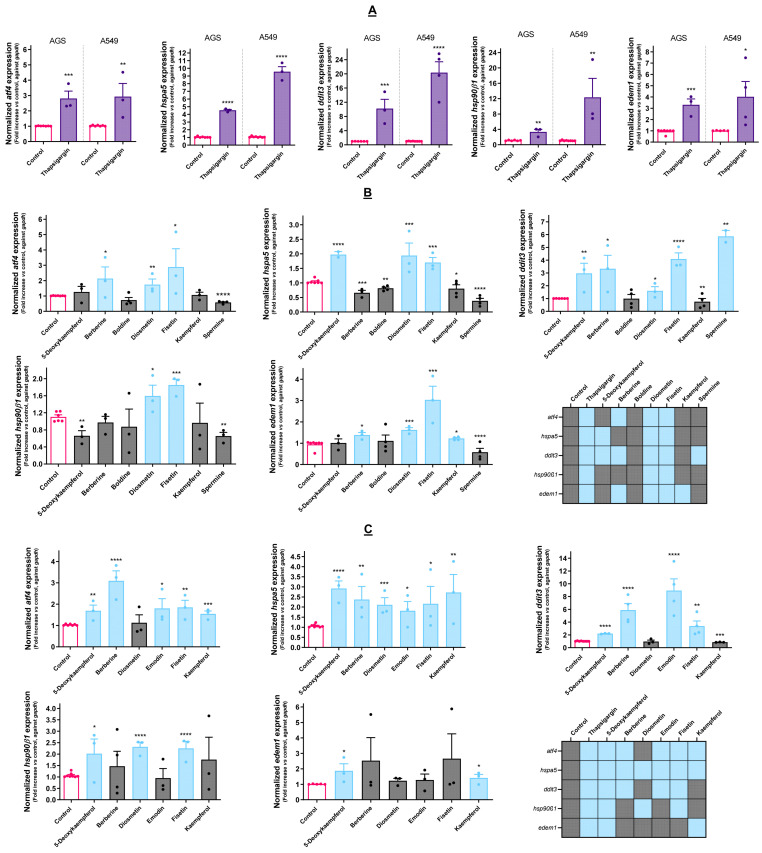
(**A**): Effect of Tg (3 µM) on the expression of UPR-related genes in AGS and A549 cells, as determined by RT-qPCR. (**B**): Effect of the selected cytotoxic molecules on the expression of UPR-related genes in AGS cells, as determined by RT-qPCR. (**C**): Effect of the selected cytotoxic molecules on the expression of UPR-related genes in A549 cells, as determined by RT-qPCR. In both cell lines, *GAPDH* was selected as a reference gene for the normalization of gene expression. Results portray the mean ± SEM of three independent assays individually conducted in duplicate. * *p* < 0.05, ** *p* < 0.01, *** *p* < 0.001, **** *p* < 0.0001.

As shown in Figure 2A, both AGS and A549 cells had a similar response in terms of *ATF4* and *EDEM1* expression when challenged with Tg, increasing the expression of these genes by about 3 to 4-fold. For the remaining genes, namely *HSPA5*, *DDIT3*, and *HSP90β1*, the response was different between the two cell lines, with A549 exhibiting a stronger response than AGS. These results show that the genes selected are adequate for assessing the UPR.

After assessing the genetic response in the context of UPR, all of the molecules selected as cytotoxic were evaluated in the same conditions. As shown in Figure 2B,C, all of the molecules were able to upregulate the expression of at least one of the target genes, with the exception of boldine in the case of AGS cells. Therefore, we hypothesized that the toxicity exerted by this molecule does not involve the UPR, and for this reason, it was dropped from the pipeline. In these cells, diosmetin and fisetin upregulated all of the analyzed target genes, while kaempferol and spermine upregulated only *EDEM1* and *DDIT3*, respectively. The action of 5-deoxykaempferol resulted in upregulated *DDIT3* and *HSPA5* expression, while berberine increased the transcription factors *ATF4* and *DDIT3* and also *EDEM1*. Concerning the A549 cells, 5-deoxykaempferol increased the expression of all of the target genes, berberine and emodin, increasing the expression of *ATF4*, *HSPA5*, and *DDIT3*. Fisetin upregulated all of the genes except for *EDEM1*. Kaempferol upregulated *ATF4*, *HSPA5*, and *EDEM1*. The diosmetin treatment resulted in the increased expression of both chaperones. 

### 3.3. Selected Molecules Impact Ca^2+^ Homeostasis and Caspase Activity

**Figure 3:**
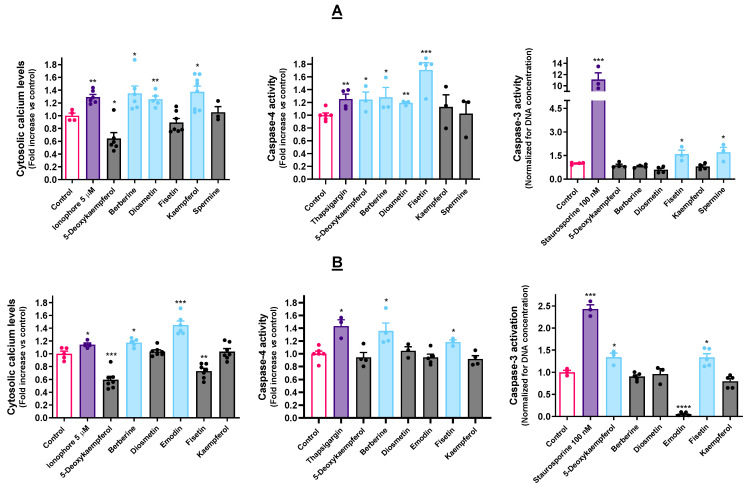
Effect of the molecules under study on the amounts of cytosolic Ca^2+^ in AGS (**A**) and A549 (**B**) cells, as determined with the fluorescent probe Fura-2/AM. Results are calculated considering F_340/505_/F_380/505_ and represent the mean ± SEM of at least three independent assays, individually performed in triplicate. Impact of the selected cytotoxic molecules activities of caspase-4 and caspase-3 in AGS cells (**A**) and in A549 cells (**B**). The displayed results represent the mean ± SEM of at least three independent experiments. * *p* < 0.05, ** *p* < 0.01, *** *p* < 0.001, **** *p* < 0.0001.

Berberine, diosmetin and kaempferol caused Ca^2+^ to flow from the ER into the cytosol on the AGS cell line (Figure 3A). Regarding the A549 cells (Figure 3B), only berberine and emodin increased the amounts of cytosolic Ca^2+^. 

Berberine elicited caspase-4 activation in both cell lines, being the only molecule that could do so in concomitance with impacting Ca^2+^ homeostasis under the selected experimental conditions. Fisetin could also significantly induce caspase-4 activation in both cell lines, while 5-deoxykaempferol and diosmetin only exerted a visible effect on the AGS cells. Regarding caspase-3 activation, fisetin was the only compound that could trigger this effect in both cell lines (Figure 3A,B). Other than this, 5-deoxykaempferol triggered caspase-3 activation in A549 cells (Figure 3B). 

### 3.4. Pharmacological Modulation of UPR Impacts Cytotoxicity of ER Stressors

**Figure 4:**
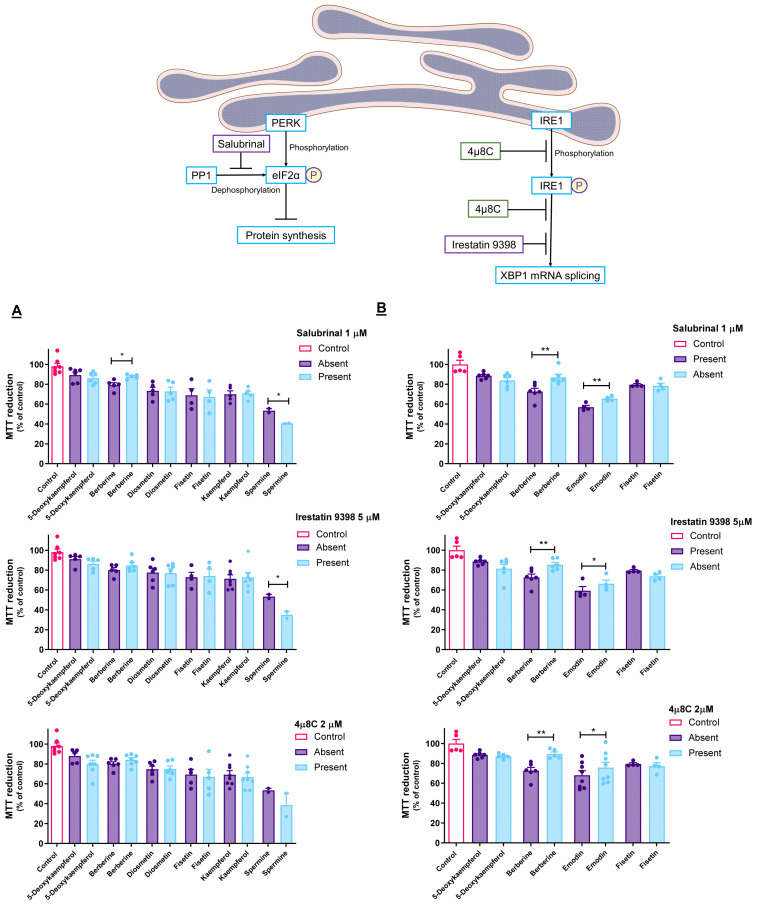
Effect of UPR inhibitors salubrinal, irestatin 9398 and 4µ8C on the impact on cell viability provoked on AGS (**A**) and A549 (**B**) cells by selected natural products. Results express the mean ± SEM of at least three independent experiments, all performed in triplicate. * *p* < 0.05, ** *p* < 0.01.

In the AGS cells, pre-incubation with salubrinal (1 µM) resulted in the reduced toxicity of berberine (Figure 4A). As for the A549 cells, the same effect was observed in the cases of berberine and emodin (Figure 4B). Berberine was the only of these molecules to be tested on both cell lines, and the results show that its effect extends to both. Furthermore, the salubrinal-induced increase in PERK phosphorylation enhances the toxicity of spermine against the AGS cells.

Unlike what was verified in the presence of salubrinal, IRE1 inhibitors failed to prevent the toxicity of berberine on the AGS cells (Figure 4A). Regarding the A549 cells, both irestastin 9398 and 4µ8C could prevent cell death induced by berberine and emodin (Figure 4B). 

### 3.5. Cytotoxic Effect of ER Stressors Is Retained in 3D Models 

**Figure 5:**
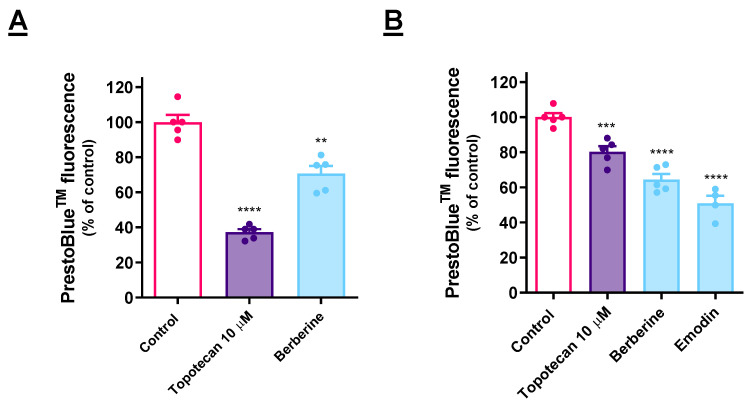
Effect of the molecules under study on the viability of AGS (**A**) and A549 (**B**) cells in 3D cell culture conditions, as determined by the fluorescence of PrestoBlue^TM^. Results are calculated considering F560/590 nm and represent the mean ± SEM of at least three independent assays, individually performed in triplicate. ** *p* < 0.01, *** *p* < 0.001, **** *p* < 0.0001.

A 3D cell culture system was implemented to test the selected compounds (Figure 5). In the AGS cells (Figure 5A), berberine retained the cytotoxic effect. In the case of the A549 cells (Figure 5B), both berberine and emodin were active in this regard. Notably, berberine and emodin present a stronger effect than that of the positive control. 

## 4. Discussion

### 4.1. Selection of Cytotoxic Molecules towards Cancer Cells

Aiming to avoid the development of anticancer molecules that lack selectivity toward cancer cells, in this work, we have only used non-toxic compounds for human non-cancer cells. It is widely reported that even though the search for molecules that do not exert undesirable side effects on healthy cells is fierce, the vast majority of attempts at the discovery of such molecules has fallen short [26].

Considering that most novel cytotoxic cancer drugs are capable of triggering an array of events that are classified as OCD, we were interested in further filtering the results from cell viability. We have chosen to evaluate the impact of these molecules in the mitochondrial membrane potential (ΔΨ_m_) since the loss of ΔΨ_m_ is involved in most processes of OCD, its dissipation being also a hallmark of ER stress, as a consequence of the upregulation of BH3-only proteins [27]. As so, molecules that have displayed selective cytotoxicity along with decreased ΔΨ_m_ have been studied in the following assays.

### 4.2. The Selected Molecules Are Capable of Triggering UPR Activation

The onset of ER stress leads to the upregulation of the expression of a battery of genes, including the genes encoding chaperones and transcription factors downstream of the activation of any of the major signaling branches of the UPR. 

**Figure 6:**
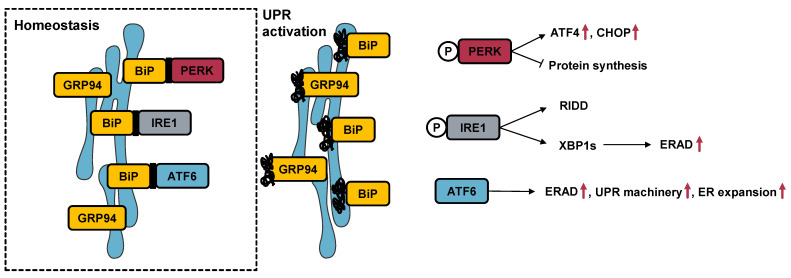
Involvement of the genes analyzed by RT-qPCR in ER stress signaling.

The *ATF4* gene encodes the activating transcription factor-4 (ATF4), a transcription factor inducible by UPR activation, specifically of the PERK signaling branch. Even though PERK signaling leads to an overall decrease in the protein synthesis rate, the enhanced translation of a discrete group of proteins, including ATF4, occurs [28]. In turn, ATF4 upregulates genes, such as *DDIT33*, which encodes the C/EBP homologous protein (CHOP). This transcription factor is pivotal in the process of ER stress-induced apoptosis, and its transcription may also be induced by IRE1 or ATF6 signaling, the other two major signaling branches of the UPR [29]. The *HSPAa5* gene corresponds to the binding immunoglobulin protein (BiP) or 78-kDa glucose-regulated protein (GRP78), while *HSP90β1* encodes the 94-kDa glucose-regulated protein (GRP94). Both genes correspond to Ca^2+^ binding proteins and are the most abundantly expressed chaperones in the ER. In fact, BiP is firmly established as the major gatekeeper of UPR activation [30]. Furthermore, the expression of both genes is known to be upregulated upon ATF6 activation [31]. Under homeostatic conditions, the mRNA encoding the transcription factor X-box-binding protein 1 (XBP1) is found in the cell in its unspliced form. IRE1 activation leads to its splicing and activation, resulting in the increased expression of genes, such as *EDEM11*, which generates the ER degradation-enhancing alpha-mannosidase-like protein 1 (EDEM1) [32]. This well-known target of XBP1 is involved in ERAD [33]. The involvement of these genes in ER stress is schematized in Figure 6.

Tg was used as a positive control for the upregulation of the aforementioned target genes in each cell line, accounting for the cell-specific differences in the cellular response, as displayed in Figure 2A. This compound has been extensively employed as a positive control for the occurrence of ER stress and the activation of the UPR ever since its characterization as a selective SERCA pump inhibitor in the early 1990s [14,15]. The SERCA pump oversees the uptake of Ca^2+^ ions from the cytosol and their storage in the ER lumen, and thus its function is essential in maintaining Ca^2+^ homeostasis in the cell. 

The obtained results indicate that 5-deoxykaempferol, diosmetin, fisetin, berberine, emodin, and kaempferol elicit the activation of one or more signaling branches of the UPR, and for this reason they were considered for the subsequent assays. Other than this, the results make clear that UPR activation by the same molecule occurs differently in each cell model, hinting at the relevance of studying each cell type individually since UPR activation depends on the ER capacity and function of the respective cell [34]. This is also ascertained by Figure 2A, evidencing that the same Tg treatment induces different expressions of the target genes in each cell line.

### 4.3. ER Stressors Disturb Ca^2+^ Homeostasis and Elicit Caspase Activation

The ER is the largest Ca^2+^ reservoir of the cell, being in charge of keeping appropriate concentrations of this second messenger. The tight regulation of these concentrations is essential to the upkeep of cellular homeostasis. The ER possesses an intricate network of transporters, channels, and pumps to govern Ca^2+^ homeostasis. The disturbances of this, leading to Ca^2+^ efflux into the cytosol, are a hallmark of ER stress [35,36]. Furthermore, this efflux leads to the dissipation of the ΔΨ_m_ [37]. For all of this, the spatial and temporal changes in this ion are of pivotal relevance when studying ER modulators. As a positive control, we used the calcium ionophore A23187, which has been observed to trigger Ca^2+^ depletion from the ER [38,39].

The literature shows that, even though OCD is inseverable from the dissipation of the ΔΨ_m_, it does not always trigger caspase activation. However, caspase activation is known as a possible downstream event of ER stress [40]. Caspase-4 resides in the ER and is activated by ER stress activators such as Tg and tunicamycin [41]. As such, Tg was used as a positive control for caspase-4 activation. In turn, caspase-4 can cleave procaspase-9, rendering it active and inducing the subsequent caspase-3 activation [42,43]. Regarding caspase-3, we used staurosporine as a positive control since it is extensively reported to induce caspase activation [24,44,45].

The fact that molecules, such as emodin, seem to trigger every hallmark of UPR activation except for caspase activation may be due to the experimental conditions here employed in what concerns, for example, time period, since it is known that caspase expression and activation can change over time [46]. Alternatively, there are reports of ER stress triggering OCD independently of caspase activation, which is a hypothesis for the mechanism of action of emodin in A549 cells, as well as other molecules that failed to induce caspase activation [47]. Similarly, free Ca^2+^ concentrations may be time-dependent, and the reason for which changes can be overlooked. Moreover, ions may be relocated to the mitochondria instead of remaining in the cytosol [48].

### 4.4. Cytotoxic Molecules Disturb ER Homeostasis at Different Levels

After showing that the cytotoxicity of the molecules under study was mediated by the ER, we were interested in detailing the UPR branch that might have been involved, specifically the PERK and IRE1 pathways. Briefly, PERK signaling results in the increased phosphorylation of the eukaryotic initiation factor 2 alpha (eIF2α), leading global protein synthesis to a halt while selectively enhancing the synthesis of proteins, such as ATF4 [49]. Salubrinal is a cell-permeable selective inhibitor of eIF2α dephosphorylation that acts by inhibiting protein phosphatase 1 (PP1). The effect of salubrinal protects cells against ER stress by preventing the halt in mRNA translation caused by phosphorylated eIF2α [50]. As mentioned before, phosphorylated eIF2α selectively upregulates the expression of ATF4, which, in turn, induces the expression of the target genes responsible for ER stress-induced OCD mechanisms, such as *DDIT3* [49,50]. Thus, it is to be expected that if cells are undergoing OCD events related to insufficient PERK signaling, salubrinal may relieve the cytotoxicity, allowing us to infer the role of this pathway in the mechanism of action of the target compounds [24].

The toxicity of berberine is shown to be decreased when enhancing PERK-mediated UPR signaling (Figure 4A). On the other hand, in the case of spermine, it is likely that enhancing eIF2α phosphorylation resulted in the increased translation of pro-apoptotic genes and, consequently, increased toxicity.

The IRE1 enzyme possesses both kinase and endonuclease activities. It is activated by transautophosphorylation and plays a dual role in UPR signaling, being able to promote cell death or survival, such as PERK. On the one hand, its endonuclease activity results in the splicing of the mRNA corresponding to the transcription factor XBP1, which promotes cell survival by inducing the expression of ER-related genes. On the other hand, it can lead to cell death through increased IRE1-dependent decay of RNA (RIDD) [6,51]. 4µ8C is capable of inhibiting both activities of IRE1 by binding both its kinase and RNAse domains, simultaneously halting mRNA splicing and RIDD [6,52,53]. Irestatin 9398, on the other hand, inhibits only its RNAse domain, thus preventing XBP1 mRNA splicing [54]. The co-incubation of the target molecules with these specific inhibitors may therefore shed light on the involvement of IRE1 signaling in their effect.

In brief, regarding the active molecules, namely berberine towards AGS cells and berberine and emodin towards A549 cells, the selective inhibitors of PP1 and IRE1 were effective in preventing cell death. These results show that the molecules induce ER stress, leading to a collective activation of at least two of the three major branches of the UPR machinery. However, these results prove the involvement of the UPR in the effects of berberine and emodin, but they do not refute the involvement of the UPR in the cytotoxic effect of the remaining molecules, as the UPR signaling branches are often redundant and can potentially be overactivated once one of them is inhibited [55,56,57].

### 4.5. Cytotoxic Effect of ER Stressors Is Retained in 3D Models

Traditional monolayer cell cultures are, to this day, a powerful and the most extensively employed tool in the discovery of drug candidates and subsequent characterization of their mechanism of action [58]. However, the surrounding environment of a cell cultured in monolayers differs considerably from an in vivo situation. These cells attach to the polystyrene surface of cell culture plates, losing their three-dimensional conformation, lacking normal cell-to-cell interactions, and growing in the absence of the extracellular matrix. The deprivation of a natural microenvironment often leads to misleading results [59,60]. Three-dimensional cell culture systems recreate the natural microenvironment of the cells more closely [61]. On monolayer cell cultures, the test subjects are proliferating cells, while, in these systems, the test subjects are spheroids that retain natural cell morphology, cell–cell and cell-matrix interactions, and include proliferating, apoptotic, and necrotic cells. The proliferating cells are present in the outer areas, whereas the core of the spheroid remains under the pressure of receiving less oxygen and nutrients and, therefore, may be in a quiescent, hypoxic, or necrotic state [60,62]. 

Berberine induced UPR activation in both of the cell models analyzed, and it was confirmed that its cytotoxic effect translates into a 3D cell model. Emodin was active specifically against the A549 cells in the previous assays, and it retained its cytotoxic potential in a 3D cell model based on this cell line.

## 5. Conclusions

The occurrence of selective cytotoxic effects among a group of natural products was described. The cytotoxicity of some of these molecules was shown to rely on the induction of ER stress. The future may provide opportunities for further studies using berberine and emodin, the most auspicious compounds, expanding the tested concentrations and increasing the diversity of employed cell models to clarify whether the potential of the molecules is broader than the types of cancer here represented.

Berberine induced the expression of UPR target genes, as well as disturbing Ca^2+^ homeostasis and promoting ER-resident caspase-4 activation. UPR activation was further confirmed upon experiments with selective pharmacological inhibitors of key UPR mediators. Emodin resulted in the increased expression of the same genes as berberine in A549 cells, namely *ATF4*, *DDIT3*, and *HSPA5*, disturbed cytosolic Ca^2+^ levels, and its toxicity was mitigated by co-incubation with UPR modulators. Relevantly, both molecules were effective in decreasing the viability of a 3D cell model of the cell lines against which they were active. 

Being active against the two evaluated cell models, berberine is ascribed as the most promising compound to develop a selectively cytotoxic drug that induces ER stress in cancer cells. 

## Figures and Tables

**Table 1:** Data relative to the selected UPR-related genes and respective primers.

Gene	Accession Number	Primers	Annealing Temperature (°C)	Amplicon Length (bp)
*GAPDH* (GAPDH)	NM_002046.6	F: AGGTCGGAGTCAACGGATTT	60	157
		R: TGGAATTTGCCATGGGTGGA		
*HSPA5* (GRP78)	NM_005347.4	F: ACTCCTGAAGGGGAACGTCT	59.5	161
		R: TTTTCAACCACCTTGAACGGC		
*DDIT33* (CHOP)	NM_001195053.1	F: AAGTCTAAGGCACTGAGCGT	59	93
		R: TTGAACACTCTCTCCTCAGGT		
*HSP90β1* (GRP94)	NM_003299.2	F: GCTCTATGTGCGCCGTGTAT	60.5	91
		R: ATCTGAGTCCACCACACCCTT		
*ATF4* (ATF4)	NM_001675.4	F: ACAACAGCAAGGAGGATGCC	60	135
		R: CCAACGTGGTCAGAAGGTCA		
*EDEM1* (EDEM1)	NM_014674.2	F: GCGGGGACCCTTCAAATCT	60	117
		R: CGGCTTTCTGGAACTCGGAT		

## Data Availability

The data generated during and/or analyzed during the current study are available in the article and Supplementary Material.

## Supplementary Materials

The following supporting information can be downloaded at: https://www.mdpi.com/article/10.3390/cancers15010293/s1, Table S1: Legend of the heatmap, containing the molecules that were tested in cell viability assays; Figure S1: Effect of a library of natural products upon AGS cell viability, as determined by MTT reduction assays; Figure S2: Effect of a library of natural products upon A549 cell viability, as determined by MTT reduction assays.

## Abbreviations

ATF4, activating transcription factor 4; ATF6, activating transcription factor 6; CHOP, C/EBP homologous protein; EDEM1, ER degradation-enhancing alpha-mannosidase-like protein 1; ER, endoplasmic reticulum; ERAD, ER-associated degradation; IRE1, inositol-requiring enzyme 1; NLRP3, NOD-, LRR- and pyrin domain-containing protein 3; OCD, organized cell death; PERK, protein kinase RNA-like endoplasmic reticulum kinase; PMA, phorbol 12-myristate 13-acetate; Tg, thapsigargin;; UPR, unfolded protein response.
