# Supplementary material for: A Survey of Naturally Occurring Molecules as New Endoplasmic Reticulum Stress Activators with Selective Anticancer Activity
Source: Cancers (Basel). 2022 Dec 31;15(1):293. doi: 10.3390/cancers15010293 (PMC9818656; doi:10.3390/cancers15010293)
Supplement: Supplementary file 1 [file cancers-15-00293-s001.zip › cancers-2086839-supplementary.pdf]

cancers

MDPI

Supplementary Material

A survey of naturally-occurring molecules as new endoplasmic reticulum stress activators with selective anticancer activity

Table S1. Legend of the heatmap, containing the molecules that were tested in cell viability assays.

	A	B	C	D	E
**1**	Control	Berberine	Cynarin	Fisetin	Homoorientin
**2**	(-)-Norepinephrine	Betanin	Daidzein	Flavanone	Homovannilic acid
**3**	(+/-)-Dihydrokaempferol	Boldine	Delphinidin	Galanthamine	Isorhamnetin-3-*O*-glucoside
**4**	3,4-Dihydrobenzoic acid	Caffeine	Diosmetin	Gallic acid	Isorhamnetin-3-*O*-rutinoside
**5**	3,4-Dimethoxycinnamic acid	Catechol	Ellagic acid	Genistein	Isorhoifolin
**6**	3-Hydroxybenzoic acid	Chlorogenic acid	Emodin	Gentisic acid	Juglone
**7**	4-Hydroxybenzoic acid	Cholesta-3,5-diene	Eriocitrin	Guaiaverin	Kaempferol
**8**	5,7,8-Trihydroxyflavone	Cinnamic acid	Eriodictyol	Herniarin	Kaempferol-3-*O*-rutinoside
**9**	5-Deoxykaempferol	Coumarin	Eriodictyol-7-O-glucoside	Hesperetin	Kaempferol-7-*O*-neohesperidoside
**10**	Apigetrin	Cyanidin	Ferulic acid	Homoeriodictyol	Liquiritigenin
	**F**	**G**	**H**	**I**	**J**
**1**	Luteolin-3-7-*di*-glucoside	Naringenin-7-glucoside	Pinocembrin	Saponarin	Tiliroside
**2**	Luteolin-4'-*O*-glucoside	Naringin	Pyrogallol	Scopolamine	Trigonelline
**3**	Luteolin-7-*O*-glucoside	Narirutin	Quercetin-3-*O*-(-6-acetylglucoside)	Sennoside B	Vanillin
**4**	Malvidin	Oleuropein	Quercetin-3-*O*-glucuronide	Silibinin	Verbascoside
**5**	Maritimein	Orientin	Quercetin-3-ß-D-glucoside	Spermine	Vicenin-2
**6**	Myricetin	*p*-Coumaric acid	Quercitrin	Sulfuretin	Vitexin
**7**	Myricitrin	Pelargonidin	Rhoifolin	Swertiamarin	Vitexin-2-*O*-rhamnoside
**8**	Myristic acid	Pelargonin	Robinin	Taxifolin	Xanthone
**9**	Myrtillin	Phloridzin	Rosmarinic acid	Theobromine	
**10**	Naringenin	Phloroglucinol	Rutin	Theophylline	

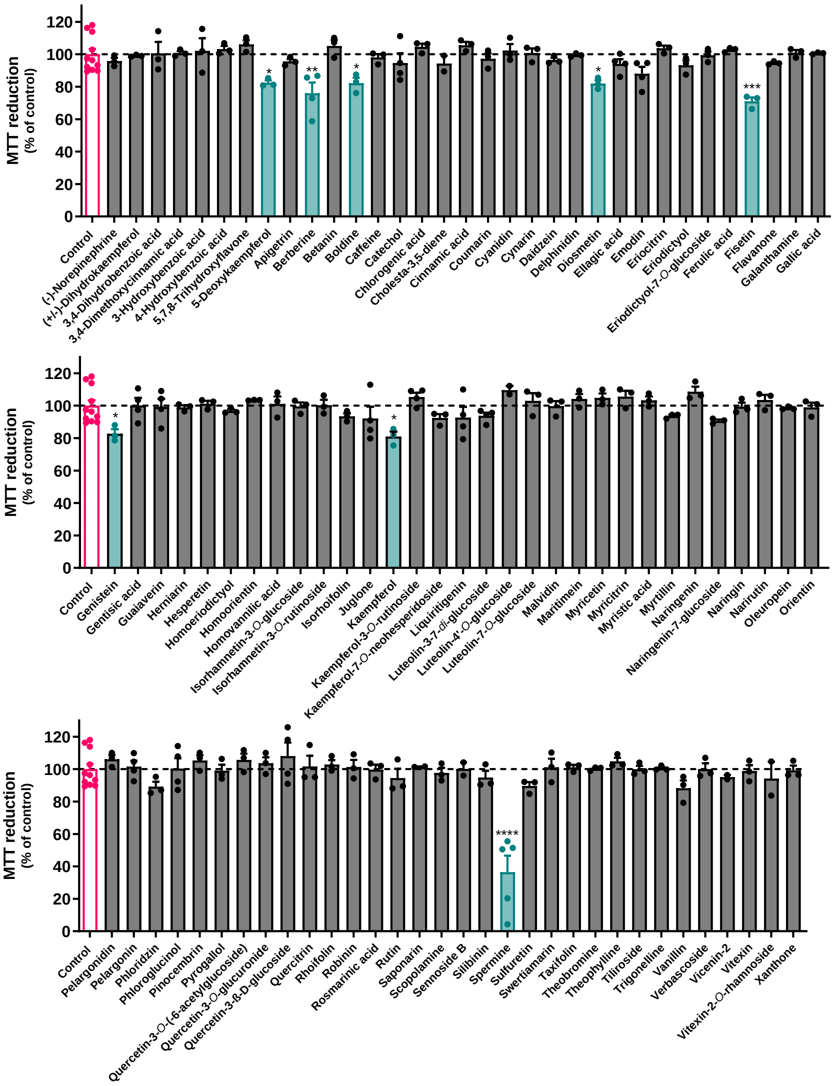

Figure S1. Effect of a library of natural products upon AGS cell viability, as determined by MTT reduction assays. All compounds were tested at 50 μM. Results correspond to the mean ± standard error of the mean and represent, at least, three independent experiments, each performed in triplicate.

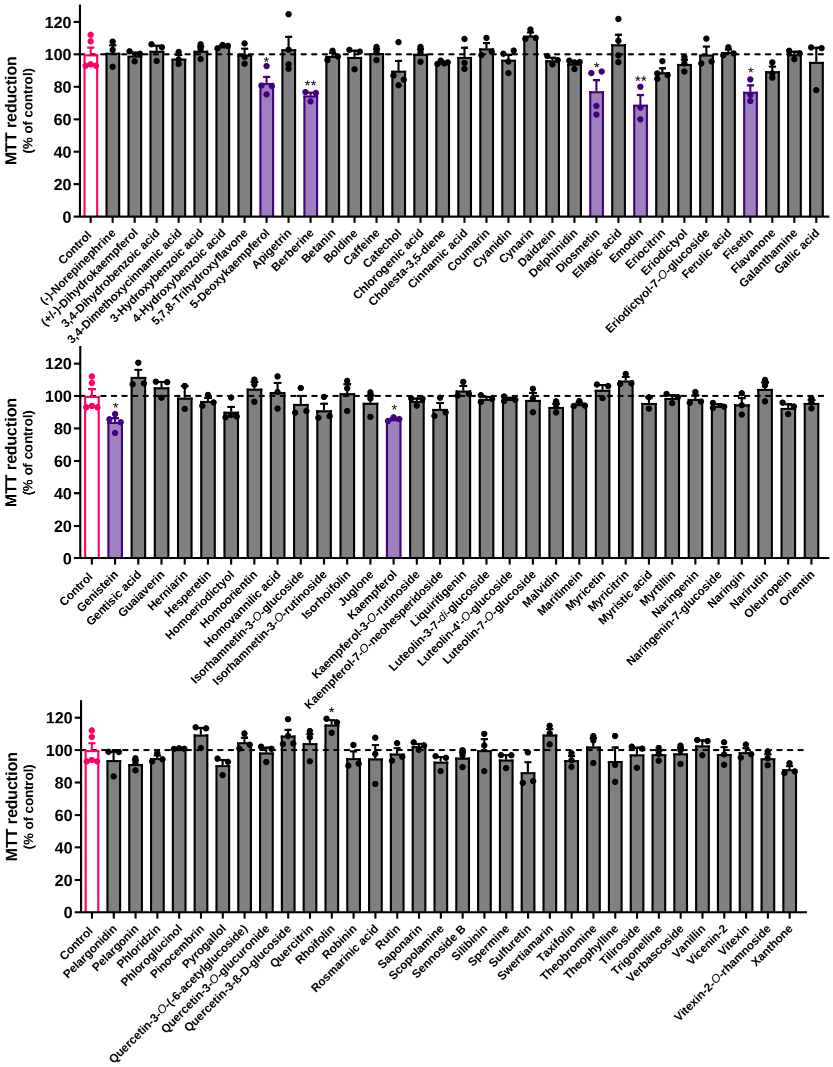

Figure S2. Effect of a library of natural products upon A549 cell viability, as determined by MTT reduction assays. All compounds were tested at 50 μM. Results correspond to the mean ± standard error of the mean and represent, at least, three independent experiments, each performed in triplicate.
